# Did Terrestrial Diversification of Amoebas (Amoebozoa) Occur in Synchrony with Land Plants?

**DOI:** 10.1371/journal.pone.0074374

**Published:** 2013-09-11

**Authors:** Omar Fiz-Palacios, Maria Romeralo, Afsaneh Ahmadzadeh, Stina Weststrand, Per Erik Ahlberg, Sandra Baldauf

**Affiliations:** 1 Systematic Biology Program, Department of Organismal Biology, Evolutionary Biology Centre, Norbyvägen 18 D, Uppsala, Sweden; 2 Evolution and Development Program, Department of Organismal Biology, Evolutionary Biology Centre, Norbyvägen 18 A, Uppsala, Sweden; Institut Pasteur, France

## Abstract

Evolution of lineage diversification through time is an active area of research where much progress has been made in the last decade. Contrary to the situation in animals and plants little is known about how diversification rates have evolved in most major groups of protist. This is mainly due to uncertainty about phylogenetic relationships, scarcity of the protist fossil record and the unknown diversity within these lineages. We have analyzed the evolutionary history of the supergroup Amoebozoa over the last 1000 million years using molecular dating and species number estimates. After an origin in the marine environment we have dated the colonization of terrestrial habitats by three distinct lineages of Amoebozoa: Dictyostelia, Myxogastria and Arcellinida. The common ancestor of the two sister taxa, Dictyostelia and Myxogastria, appears to have existed before the colonization of land by plants. In contrast Arcellinida seems to have diversify in synchrony with land plant radiation, and more specifically with that of mosses. Detection of acceleration of diversification rates in Myxogastria and Arcellinida points to a co-evolution within the terrestrial habitats, where land plants and the amoebozoans may have interacted during the evolution of these new ecosystems.

## Introduction

The supergroup Amoebozoa is a major division of eukaryotes, closely related to the clade including animals and fungi (Opisthokonta [1]). Recent molecular studies divide Amoebozoa into two major subclades, Lobosa and Conosa, with a possible third lineage, Breviatea, (*e.g. Apusomonas*) as sister to them both [2,3]. Lobosa is further divided into two subdivisions: Discosea (e.g. *Vanella*) and Tubulinea (*e.g. Arcella*), and Conosa is subdivided into three: Variosea (e.g. *Filamoeba*), Archamoebea (*e.g.* *Entamoeba* ), and Mycetozoa or slime molds (*e.g.* *Dictyostelium* ).

Amoebozoan diversity has been described mostly from rivers, lakes and marine environments. However, arcellinids (Tubulinea) mostly occur in peatlands, freshwater habitats and temperate forest soils, while the amoebozoan slime molds (Mycetozoa) are mostly known from terrestrial soils from forested areas around the world [4–7]. Several facts suggest an early divergence of Amoebozoa in marine habitats: the presence of flagella in a number of species, the early branching phylogenetic position of amoebozoan marine lineages and the fact that the earliest amoebozoan fossils (742-770 Mya) are from marine environments [8,9]. Based on these facts, it has been suggested that Amoebozoa originated in marine environments and later expanded into terrestrial niches as land plants diversified [9].

The two terrestrial amoebozoan lineages with the largest number of described species are Arcellinida (Tubulinea) and Mycetozoa (Dictyostelia + Myxogastria). Both groups consist of predators whose prey are bacteria and single-celled fungi such as yeasts. These terrestrial amoebas tend to live in habitats that are rich in organic matter, which could not have existed before the colonization of land by plants [9]. Together these groups of terrestrial amoebas are among the most abundant eukaryotic soil microbes and they play important roles in the soil, such as enhancing soil fertility through nutrient mineralization [10].

The taxonomy of Amoebozoa has a problematic history [11,12]. As a result there remains a great deal of confusion and uncertainty regarding their higher-order phylogeny, and different criteria are strongly defended [11,13–15]. Although molecular data are helping to resolve some of these relationships, these data are still scarce in terms of both taxon sampling and number of genes [14,16]. However, there is a strong consensus on the monophyly of Amoebozoa as a supergroup and a growing consensus for it consisting of two major divisions, Lobosa and Conosa [3].

A scarcity of relevant fossils has also made it difficult to interpret amoebozoan evolutionary history. The oldest and most widely accepted amoebozoan fossil is a vase-shaped microfossil (VSM) assigned to the division Lobosa and dated from 742–770 Mya [17], i.e. the Cryogenian Period of the Neoproterozoic [18]. Meanwhile, molecular reconstructions have dated the origin of modern diversification in Amoebozoa (crown age) from between ca 850-1250 Mya [19–21]. However the latter reconstructions were focused on estimating earlier divergence times, i.e. for the major groups of eukaryotes, rather than being directed specifically at Amoebozoa and the diversity within it. This is the case for most protistan lineages and molecular clock studies have only recently been applied specifically to reconstructing divergence times in select groups of protists [22]. Dating studies focused on the origin and diversification of major groups within Amoebozoa has received even less attention, due in part to their phylogenetic uncertainty and poor (and young) fossil record. Nonetheless, divergence time reconstruction in protists is crucial if we want to understand major events in the early evolution of eukaryotes [22].

In this study we use molecular data for a taxonomically broad species sampling to reconstruct the divergence times in Amoebozoa in order to investigate the timing and pattern of diversification of the major groups within it. To do this we first explore different calibration schemes, substitution models, genes, fossil assignment schema, and dating methods, as well as incorporating dating estimate uncertainty based on confidence intervals [23]. Two separate data sets are used, one consisting of six proteins (actin, alpha-tubulin, beta-tubulin, EF1a, eRF3 and RPB1) and one of 18S ribosomal DNA (18S rDNA) sequences. The clock is calibrated using four widely accepted fossils from other supergroups (plants, fungi and animals), and the information retrieved by fossil cross-validation with Bayesian methods is explored. In light of these results, we discuss possible scenarios for the colonization of land by Amoebozoa and the relationship of these events to the invasion of the terrestrial habitat by green plants.

## Materials and Methods

### 2.1: DNA isolation, PCR amplification and sequencing

Three new sequences for RPB1 and eRF3 (Table S1) were generated by PCR from total genomic DNA. DNA extraction followed the protocol of Romeralo et al. [24], with cell lysates used directly for PCR amplification. The newly designed primers are AF (CAAGAGTGTCCGGGNCAYTTCGG) and DR (GTTCATCTCGTCNCCRTCRAARTC) for RPB1 and 2F (AAGGGTAAGACTGTNGARGTNGG) and 1R (ACGTTGACCACYTTNCCRAANGC) for eRF3. PCR conditions were as follows: 94^°^C, 5 minutes followed by 35 (eRF3) or 30 cycles (RPB1): 94^°^C, 1 minutes; 55^°^C (eRF3) or 50^°^C (RPB1), 1 minute; 72^°^C, 1 minute and then a final elongation 72^°^C, 10 minutes. All PCR reactions were done using ‘illustraTM puReTaq Ready-To-Go PCR Beads’15 (GE Healthcare, Solna, Sweden). PCR products were separated on 1.5% agarose gels, and individual bands of adequate size excised from the gel and purified with ‘QIAquick Gel Extraction Kit’16 (Qiagen, Crawley, UK) according to the manufacturer’s protocol (‘Gel Extraction Spin Protocol’). Extracted gel bands were cloned with the ‘TOPO TA Cloning^®^ Kit’ (Invitrogen, Stockholm, Sweden). Screening of white colonies was for RPB1 done with the internal primers AF and RpB385R (GTTAGGCTCAGCNGTHATNACNGT).

Postcloning PCR was done as follows: 94^°^C, 10 minutes followed by 30 cycles: 94^°^C, 1 minute; 60^°^C (eRF3) or 55–60^°^C (RPB1); 1 minute and 72^°^C, 1 minute and a final elongation at 72^°^C, 7 minutes. Sequencing was done by Macrogen Inc. (Seoul, Korea) on an ABI 3730XL using the primer pair T7promoter/M13R-pUC(-40) (TAATACGACTCACTATAGGG and CAGGAAACAGCTATGAC). Sequences were edited and assembled into contigs using the Pregap4 and Gap4 modules of the Staden package version 4.1 [25]. Consensus sequences were translated into amino acids using the program ‘Six Frame Translation of Sequence’ by HGSC (http://searchlauncher.bcm.tmc.edu/seq-util/Options/sixframe.html). Sequences were edited and assembled into contigs using Sequencher 3.0 (Gene Codes Corp.).

### 2.2: Phylogenetic analyses and divergence time estimations

The three new sequences were combined with publicly available sequences from various databases in order to build two matrices (Table S1). The first matrix includes 17 amoebozoan and 15 outgroup taxa for six proteins (actin, alpha-tubulin, beta-tubulin, EF1a, eRF3 and RPB1) with the sampling strategy of including all species with sequences available for at least two of these proteins. The second matrix consists of 72 Amoebozoa and 14 outgroup taxa for 18S rDNA, for which only unambiguously aligned regions were included (Table S1).

Individual molecular markers were aligned separately using MUSCLE v 3.8 [26] and ambiguously aligned regions masked by hand. The resulting alignments were also assembled by hand into a combined matrix. The final matrices consist of 2289 aligned amino acid positions for the 6-protein matrix and 1140 nucleotide positions for the 18S rDNA matrix. Nexus formatted files of the two matrices are available as supporting material (Nexus file S1 and Nexus file S2). Maximum likelihood analyses consisted of 1000 bootstrap replicates followed by optimal tree searching using RAxML v 7.04 [27], under the WAG and GTR substitution models for protein and DNA sequences, respectively.

Four widely accepted fossils were used to constrain the resulting trees: land plants at 423 myr [28], seed plants at 310 myr [29], Metazoa at 580 myr [30,31] and Ascomycetes at 400 myr [32]. Based on uncertainty of assignment of the ascomycete fossil [33] two alternative positions were explored, one at the stem node and one at the crown node of Ascomycetes. All four fossils were first tested for consistency by fossil cross-validation (SSx [34]) using the 6-protein data set and BEAST v 1.5.4 [35] with a uniform or a normal distribution for fossil age assignment and the WAG model of amino acid substitution.

Full reconstruction of divergences times was carried out separately using BEAST v 1.5.4 [35] and MCMCtree [36]. BEAST analyses used the WAG (for protein) and GTR and HKY (for DNA sequences) models of substitution, and were run under an uncorrelated lognormal (UCLN) model that allows different evolutionary rates in different branches of the tree. The effect of substitution rate heterogeneity on age reconstructions was explored for protein (WAG + gamma) and DNA datasets (GTR + gamma and HKY + gamma). A uniform distribution for fossil age assignment was applied with a hard upper and lower bound equal to the fossil age and fossil age plus 10 myr, respectively, and a maximum age for the root of the tree of 3000 myr. A soft upper bound was used exclusively for calibrating the land plant crown age in order to incorporate the age uncertainty associated with this node. Based on the Rhynie chert fossil a lognormal distribution with an offset of 423 myr and a 95% upper limit of the distribution of 440 myr was applied. In order to explore a potential over estimation of the age assigned to a given node, analyses were run using a normal distribution centered on the fossil age for all fossil age assignments and a standard deviation of 10 myr. All analyses were run for 50 x 10^6^ generations, of which 10 million were discarded as burnin.

Molecular clock analyses were performed using MCMCTree [36] under the independent rates model that allows different evolutionary rates in different branches of the tree. The gamma prior on the overall substitution rate consisted of the fixed shape parameter (a = 1) to represent a diffuse prior and the scale parameter (b = 45). Branch lengths were calculated using the WAG model in CODEML [36]. Uniform distributions were applied to all fossil calibration with a soft upper and lower bound equal to the fossil age and fossil age plus 10 myr. Results were sampled every 75 iterations for a total of 15 million iterations, with 1.5 million discarded as burn-in.

### 2.3: Diversification rate shifts

Estimation of diversification rate shifts was performed using the 18S rDNA chronogram and species numbers estimated from various sources (Table S2) with the MEDUSA software implementing birth and death models [37]. An estimated number of species was assigned to every genus in the tree except for the five arcellinids, eight dictyostelids, and eleven myxogastrids (Table S2). The latter species-rich lineages were first reduced to two representatives each (always including the most ancient node) and then the total number of species in the lineage was split equally between the two subdivisions. In order to explore the possible effects of sampling bias and high number of recent cladogenic events (oversampling of nodes of interest), analyses were also performed with the total number of species in each clade divided among the total number of representatives in the tree (five, eight, and eleven, Table S2). Sixteen species number estimates were excluded due to their uncertain phylogenetic assignment (Table S2).

## Results

### 3.1: Phylogenetic reconstructions

**Figure 1:**
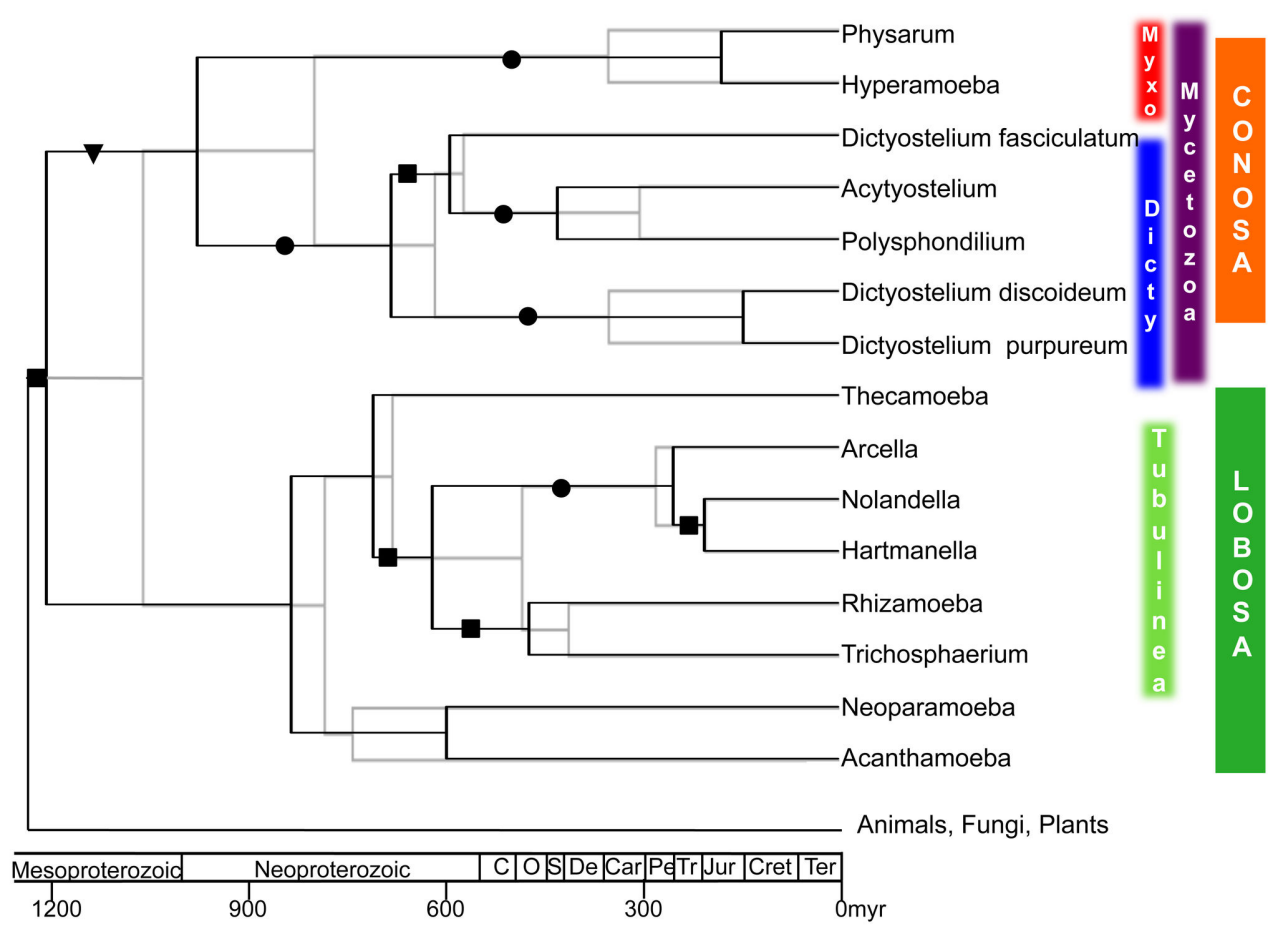
Diversification dates for Amoebozoa based on a 6-protein data set. The two trees shown are superimposed chronograms of Amoebozoa based on analyses of a 6-protein concatenated data set using BEAST [35], with the WAG model of substitution and calibrated either with two opistokont fossils (grey tree) or two opisthokont and two plant fossils (black tree). Taxonomic circumscription is indicated on the right where Myxo stands for Myxogastria and Dicty for Dictyostelia. The geological time-scale is depicted at the bottom; acronyms within the Phanerozoic are C for Cambriam, O for Ordovician, S for Silurian, De for Devonian, Car for Carboniferous, Pe for Permian, T for Triassic, Jur for Jurassic, Cret for Cretaceous and Ter for Tertiary. Bootstrap support (BS) values are indicated by black circles (BS>95%), black squares (BS>85) and black triangle (BS>75).

Phylogenetic analysis of a 6-protein dataset reconstructs the main groups of interest here, particularly Myxogastria plus Dictyostelia (Mycetozoa) and a monophyletic Tubulinea including the Arcellinida (Figure 1 [3]). The only inconsistencies with respect to generally accepted amoebozoan phylogeny are the position of *Entamoeba* , which is found embedded within Tubulinea instead of sister group to Mycetozoa and the position of *Phalansterium*, which breaks the monophyly of Amoebozoa (data not shown [3,38]). Therefore, due to the extreme length and phylogenetic instability of their branches, which have been noted previously [3,38], *Phalansterium* and *Entamoeba* were excluded from the protein data in further analyses.

**Figure 2:**
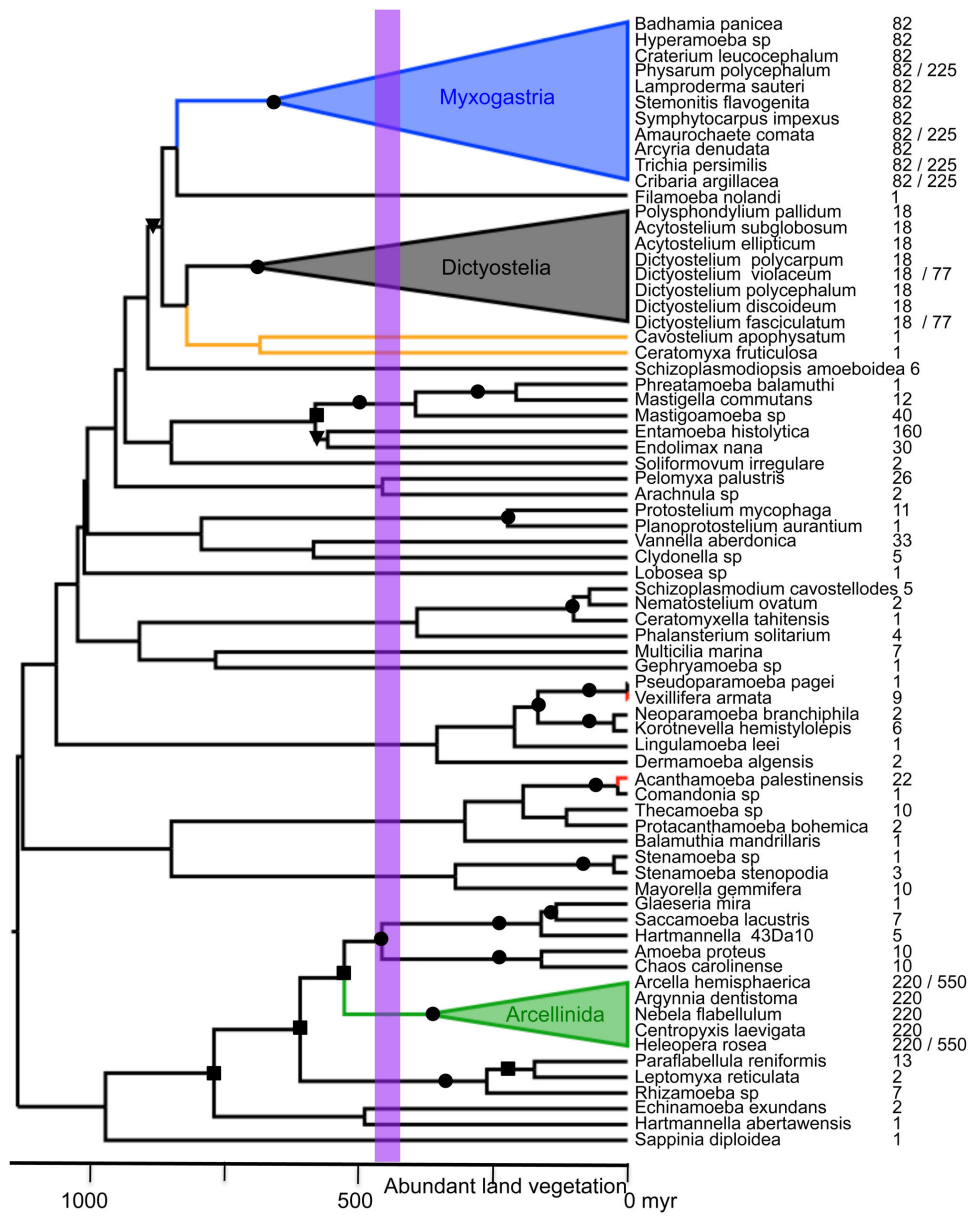
Diversification rate shifts in Amoebozoa based on an 18S rDNA chronogram. The tree shown was derived and dated using BEAST [35] with the HKY substitution model. Colored branches indicate significant rate shifts detected by the birth-death model of MEDUSA [37]: red branches, green triangle (arcellinids) and blue triangle (Myxogastria) indicate faster rates and an orange branch indicates a single terminal clade with slower rates ( *Acanthamoeba* ). Alternative coding of the number of species for each terminal is indicated with a slash (see section 2.2). Violet vertical bar indicates the time span between the initial colonization of land by plants to the origin of the terrestrial ecosystems [50]. Bootstrap support (BS) values obtained from a maximum likelihood analysis using RAxML v 7.04 [27] are indicated by black circles (BS>95%), black squares (BS>75) black triangles (BS>50).

Since there are few Amoebozoa for which substantial protein sequence data are available, we also constructed a more taxonomically detailed phylogeny using 18S rDNA. This data set includes many more taxa than the 6-protein data set, but still roughly reconstructs all major amoebozoan groups (Tubulinea, Archamoebae) and especially those of interest here, i.e. Dictyostelia, Myxogastria and Arcellinida (Figure 2). As previously noted the protostelid slime molds are shown to be polyphyletic (Figure 2 [5,39]). Both trees are consistent with recent findings on amoebozoan phylogeny pointing to the difficulty of recovering higher-level relationships with the exception of Tubulinea, Archamoebae and Myxogastria [38].

### 3.2: Calibration parameters

Before calibrating the tree, we tested the consistency of the individual fossils with these data and the effects of various model parameters on date estimates. Fossil consistency was evaluated using fossil cross-validation, which measures the accuracy with which single-fossil calibrated trees reconstruct known fossil dates [34]. First we examined dating alternatives for the most ambiguous fossil here, the ascomycete fossil, which can be assigned to either the ascomycete crown or stem depending on how the fossil is interpreted [33]. Placing the fossil at the ascomycete crown node results in smaller disagreement between dates predicted by trees calibrated with the fungal versus metazoan fossils (Figures S1A and S1B) than when the fossil is placed at the ascomycete stem node (Figures S1C and S1D). Using the ascomycete stem node for this fossil also retrieves substantially younger ages across the timetree, a “pull to the present” that results in large discrepancies between reconstructed dates and the fossil record (Table S3). Therefore this fossil was placed at the ascomycete crown node in all further analyses.

Fossil cross-validation also shows that trees calibrated with the land plant (423 myr) or seed plant (310 myr) fossils give inaccurate age predictions for the remaining fossils (high SSx values, Figure S1). These discrepancies are further exacerbated when using a uniform as opposed to a normal distribution for fossil assignment (Figure S1). Analyses using only the two plant fossils also recover unrealistically old ages, for example placing the root of eukaryotes at a mean age of 1748 Mya (95% highest posterior density or HPD = 1202-2659, Table S1 [20]). Therefore all further reconstructions used parallel calibrations, one with all four fossils and the other with the two opisthokont fossils alone (400 and 580 myr).

Other than differences with the plant fossils, similar results were obtained in analyses of the protein chronogram using a normal or uniform distribution (Figure S1). For example, the amoebozoan mean crown age ranges from 1125–1437 Mya using a normal distribution (95% HDP = 705-1650 Mya, Table 1) versus 1041-1224 Mya with a uniform distribution (95% HPD = 755-1805 Mya, Table S3). Since our fossil assignment using a normal distribution implies a violation of the minimum age given by the fossil itself and because results are otherwise not significantly different from the ones obtained using a uniform distribution, we use uniform distributions in all further analyses.

**Table 1:** Divergences times reconstructed from 6-protein and 18S rDNA data sets using different calibration schemes and models.

**Fossil calibration**	**Tree Root**	**Amoebozoa**	**Mycetozoa**	**Dictyostelids**	**Arcellinids (stem**)
6-proteins					
4 fossils	1493 (1126-2104)	1224 (862-1693)	990 (603-1378)	691 (306–842)	254 (190–655)
4 fossils gamma	1710 (1143-2093)	1145 (934-1767)	912 (598-1421)	731 (288–794)	366 (267–806)
4 fossils M	1418 (1091-1877)	1193 (909-1565)	972 (704-1312)	611 (412–864)	445 (284–671)
580+400	1420 (973-1839)	1073 (757-1450)	809 (505-1164)	624 (278–760)	284 (177–570)
580+400 gamma	1131 (930-1865)	1041 (753-1566)	597 (476-1239)	341 (247–699)	403 (239–716)
580+400 M	1358 (988-1878)	1142 (807-1588)	927 (609-1335)	572 (351–893)	431 (223–680)
18S rDNA					
580 + 400 HKY	1355 (1127-2376)	1123 (1035-2108)	857 (620-1202)	699 (293–859)	522 (436-1121)

Divergence times (in million years) are for crown groups using the WAG amino acid substitution model, Uniform distribution and an ascomycetes crown node fossil placement unless otherwise stated. Values for the 95% HPD (highest posterior density) are indicated in parenthesis. Fossil combinations correspond to four fossils or the metazoan and fungal fossils alone (580+400 myr). Dates were derived using BEAST [35] except for those indicated as “M”, which were derived using MCMCtree [36].

**Figure 3:**
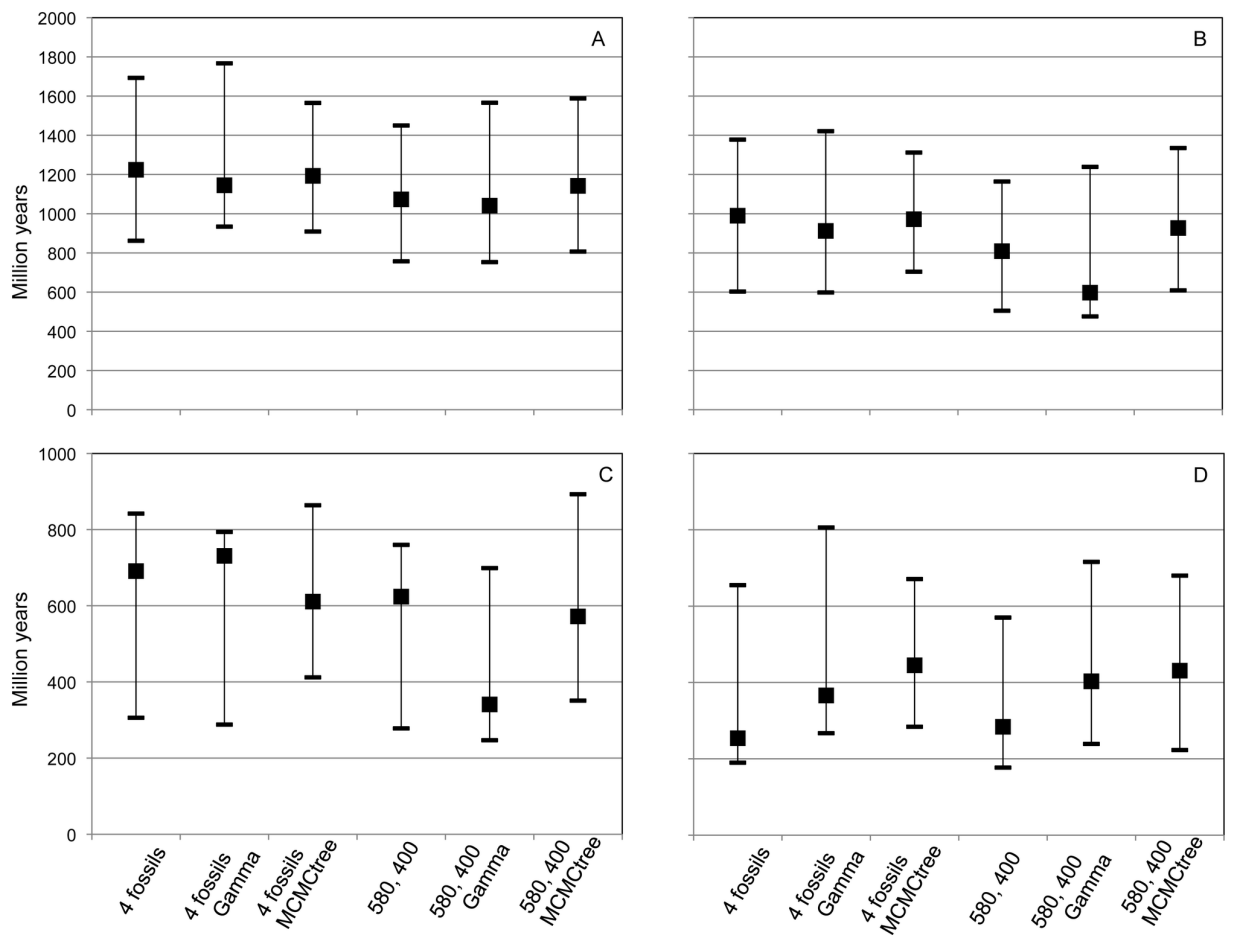
Reconstructed node ages and their 95% HPD distribution based on a 6-protein phylogeny. Reconstructed ages and 95% highest posterior density (HPD) from a 6-protein data set under different fossil constraints (four or two fossil -580-400 myr), models (WAG, with or without a gamma rate correction) and programs (BEAST or MCMCtree) for the following nodes: A) crown Amoebozoa B) crown Mycetozoa C) crown Dictyostelia and D) stem Arcellinida. Note the different scales among the plots. The exact values for nodes and HPDs are given in Table 1.

Divergence times were estimated separately using the software BEAST (Drummond and Rambaut, 2007) and MCMCtree (Yang, 2007). Dates estimated with the 6-protein data set using a uniform distribution and the ascomycete crown node show very similar results between the two methods, with mean age differences ranging from 18 to 191 myr (Table 1). There is also little difference between the two methods when using all four fossils or the two opistokont fossils alone (Table 1). Larger differences in date estimates were seen when incorporating a correction for heterogeneity of substitution rates using a gamma distribution (Table 1 and Figure 3). Although the latter analyses do not show a large difference in age ranges (95% HPD), these analyses give particularly young mean ages for the dictyostelid and Mycetozoa crown when the trees are calibrated with only two fossils (Table 1 and Figure 3).

For the 18S rDNA chronogram the largest differences in reconstructions arise from the use of all four fossils versus the two opistokont fossils alone (Table S3). Four fossil reconstructions with 18S rDNA also give much older and often unrealistic ages as opposed to two fossil calibrations. This is particularly true for deep branches; for example for the eukaryote root the mean age reconstructed from 18S rDNA with four fossils is nearly the same as that estimated for the amoebozoan crown (2346-2597 Mya and 2141-2454 Mya, respectively, Table S3). On the other hand, the nucleotide substitution model used does not appear to make a large difference, as similar ages are reconstructing using either the GTR and HKY models with or without a gamma correction (Table S3). Although ages reconstructed with 18S rDNA using the two opistokont fossils are more similar to those obtained in the 6-protein analyses, the confidence intervals (95% HPD) from 18S rDNA are always broader (Table 1 and Table S3).

### 3.3: Divergence time estimates

Using a uniform distribution and the ascomycete crown node, the origin of the modern radiation of Amoebozoa is placed at 1041-1224 (753-1767) Mya based on different analyses of the 6-protein data set. These results show little variation between analyses based on different substitution models (i.e., WAG model with and without a gamma correction) or different fossil calibration schemes (2- or 4-fossil calibration; Table 1). The ascomycete crown node was therefore used in the 18S rDNA as a lower bound.

Of the terrestrial clades of interest here, the estimated age for the mycetozoan crown (Dictyostelia + Myxogastria) is 597-990 (476-1421) Mya based on the 6-protein data set calibrated with four fossils and 857-1183 (433-1705) Mya based on 18S rDNA calibrated with two opisthokont fossils (Table 1 and Table S3). It is noteworthy that without a gamma correction the age range for this node narrows substantially to 809-990 (505-1378) Mya for the 6-protein data (Figure 3). For the Dictyostelia, the two data sets analyzed here find a crown age of 341-691 (247–893) Mya and 596-786 (233-1072) Mya, respectively for 6-proteins with four fossils and 18S rDNA with two opisthokont fossils. The age range for this node decreases to 572-691 (278–893) Mya for the 6-proteins data analyzed without a gamma correction (Figure 3).

The crown age for Myxogastria can only be estimated with the 18S data, which finds an age of 661-823 (416-1291) Mya (Table S3). Myxogastria is contained within the mycetozoan clade and therefore these reconstructed ages for Myxogastria are in agreement with the 6-protein analyses of Mycetozoa (see above). Finally, the Arcellinida stem age ranges between 254–445 (190–806) Mya for 6-protein data with four fossils and 522-1039 (347-1371) Mya based on 18S with two opistokont fossils (Table 1 and Table S3). In this case when gamma correction is not taken into account the mean age range is still the same (254-445 Mya) but the upper limit of confidence intervals decreases by more than 100 myr (95% HPD’s = 190-680 Mya; Figure 3)

### 3.4: Diversification rate shifts

Diversification rate shift analysis utilized the method of Alfaro et al. (2009) as implemented in the software MEDUSA. This method detects clades exceptionally different in size, whether species-rich or species-poor, together with significant diversification rate shifts that lead to those clades. The 18S rDNA chronogram reconstructed using HKY was used for this analysis because ages reconstructed under this model were more in agreement with those reconstructed from the 6-protein data set (see above).

Rate shift analyses show the Arcellinida and the Myxogastria to be exceptionally species-rich clades (ΔAIC > 4; Figure 2) with diversification rates of 0.053 and 0.057, respectively. Both values are significantly higher than the estimated background diversification rate of 0.025. Since these analyses are based on sisterhood relationships the results should not be affected by phylogenetic uncertainty since these clades and their sister clade relationships are statistically supported here (Figure 1 and Figure 2). Two terminal clades represented here by *Acanthoamoeba* and *Vexillifera* , were also found to show high rates of diversification (0.21 and 0.34, respectively) for recent times (<35 Mya), while a clade containing two protostelids shows a low diversification rate (0.007; Figure 2). Analyses reducing the three species-rich lineages to two representatives each recovered the same rate shift patterns as analyses including the total number of representatives (Figure 2). Therefore sampling bias and number of recent cladogenic events do not seem to affect these results.

## Discussion

### 4.1: Origin of Amoebozoa and major groups within it

We have performed exhaustive analyses to reconstruct divergence times within Amoebozoa using multiple genes, broad species sampling and a range of calibration schemes. We find that ages retrieved from a 6-protein data set are generally congruent among different reconstruction schemes (Table 1). There is also substantial agreement between our protein and 18S rDNA reconstructions when the trees are calibrated using the two fossils phylogenetically closest to Amoebozoa, i.e. a metazoan and a fungal fossil (Table 1). Based on these results, we find an age for the supergroup Amoebozoa of over 1000 myr (1041-1224 myr from different approaches; Table 1). This age is congruent with previous molecular estimates of 859-1250 Mya [19–21] and also congruent with the growing number of fossil records from the Late Mesoproterozoic that are being assigned to crown eukaryotes [9].

Within the Amoebozoa, two major groups are strongly reconstructed here by both molecular data sets. These are, the Mycetozoa and Tubulinea, which include the major groups of interest here, the Dictyostelia, Myxogastria and Arcellinida (Figure 1 and Figure 2). Our molecular dating places the basal node of Mycetozoa (Dictyostelia + Myxogastria) at around 597-990 Mya and that of Dictyostelia at around 341-691 Mya while the stem node of Arcellinida at around 254-522 (Figure 3). These results represent the first estimates for Myxogastria and Arcellinida, while our estimate for Dictyostelia is in agreement with previous estimates of 600 Mya for the crown age of the group [39].

### 4.2: Colonization of the terrestrial habitat by Amoebozoa

Our node date of 597-990 Mya for Mycetozoa, which are almost exclusively known as terrestrial amoebas (Figure 2), substantially predates the first evidence of land plants in the fossil record, which is placed in the Ordovician (ca. 465 Mya [40–42]). This plant fossil record consists of spores, which are produced in abundance by their parent plants and have a good preservation potential, suggesting that this estimated date for the first appearance of plants on land is likely to be fairly precise. This date is also consistent with molecular dating using chloroplast DNA and plastid proteins (454-490 Mya [43,44]). Although some molecular studies place this node slightly earlier (~600 Mya [45,46]), this still post-dates our estimated dates for the origin of Mycetozoa. Thus we find strong evidence that the Mycetozoa originated before plants first appeared on land.

Given the deep age for the amoebozoan crown group that we find here (1041-1224 myr, Figure 3) and the fact that most fossils of this age are marine [8], the most probable scenario for the early evolution of Amoebozoa is a marine origin. This is also consistent with the fact that the oldest fossil record of Amoebozoa is from marine sediments (742-770 myr [17]). In addition, some Amoebozoa clades are found exclusively in marine environments [47].

Following this marine origin, our results indicate multiple invasions of land by Amoebozoa into both freshwater and terrestrial habitats. For freshwater habitats, there appear to have been at least three independent colonization events involving three different amoebozoan groups: Amoebidae (e.g. *Chaos*), Hartmannellidae (e.g. *Saccamoeba*), and Vannellidae (e.g. *Vannella*; Figure 2). For terrestrial habitats we find evidence of at least two independent colonization events, at least once each by Arcellinida and Mycetozoa (Figure 2). Thus invasions of non-marine habitats appear to have happened frequently across the phylogeny of Amoebozoa and at a number of different points in time. At least some of these invasions appear to have occurred early in the evolution of the terrestrial habitat based on both the age of the two major lineages of Mycetozoa (Figure 2), Myxogastria and Dictyostelia, and the fact that both lineages consist almost exclusively of terrestrial amoebas.

The two amoebozoan lineages that appear to represent independent land colonization events, the Mycetozoa and Arcellinida (Figure 1), also include or comprise the two most species-rich amoebozoan clades, the Myxogastria and arcellinids. Diversification rates in both of the latter taxa are estimated to be more than twice the background rate of Amoebozoa (0.053 and 0.057, respectively, versus a background rate of 0.027, Figure 2). Thus terrestrial colonization in Amoebozoa also appears to be correlated with two ancient diversification rate shifts in two very different and quite distantly related taxa (Figure 2). Interestingly, similar rate shifts are not seen in any other marine or freshwater clade nor in terrestrial Dictyostelia, that show a background diversification rate.

### 4.3: Scenarios for the colonization of land by amoebozoa

Amoebas are known to be important contributors to nutrient recycling in terrestrial soils [10,48]. This, together with our dating and diversification results suggest a scenario whereby amoebas participated in an initial enrichment of these soils prior to the colonization of land by plants. However, this early appearance of amoebas on land, in both freshwater and soil habitats does not appear to coincide with an acceleration in their rate of diversification. Instead most cladogenesis in Mycetozoa appears to have occurred after the Devonian (ca. 419-359 Mya), which would be well after the colonization of land by plants (Figure 2). The origin of the modern radiation of Arcellinida also seems to be well after the origin of land plants. Thus various lineages of Amoebozoa may have colonized land early, and then diversified along with an increase in diversity of terrestrial habitats.

Regardless of the exact timing of the origin of land plants, their emergence as a major component of the terrestrial ecosystem can be securely dated on the basis of plant macrofossils, spore diversity, and the appearance of new soil types [49,50]. These studies show that this colonization was a gradual process beginning around ca. 500 Mya [50]. However, terrestrial habitats must have expanded dramatically with the evolution of tree-sized plants during the Devonian (ca. 419-359 Mya) and the diversification of different groups of vertebrates and arthropods (Figure 2 [49–51]). This appears to coincide with major cladogenesis in Mycetozoa, suggesting that these terrestrial amoebae diversified alongside land plants, expanding to fill various niches as they were created.

Thus, our temporal reconstructions support the suggestion of Porter et al., that the diversification of terrestrial amoebas was linked to the differentiation of terrestrial habitats rich in organic matter [9]. Such organic matter constitutes a rich habitat for the primary food resource of bacterivorous amoebas such as dictyostelids, myxogastrids and arcellinids. Among these three groups, myxogastrids are particularly and extremely cosmopolitan having a wide habitat range including coarse woody debris, ground litter, the bark surface of living trees, dung, soil, and aerial litter [52]. However, the other two well-studied groups of Amoebozoa examined here, dictyostelids and arcellinids, have more restricted habitat preferences. Dictyostelids are mostly associated with the soil/litter microhabitat of forest floors [48], while arcellinids are mostly associated with mosses [53]. Therefore even though dictyostelids and myxogastrids may have colonized land before and arcellinids after the land plants, their subsequent diversifications seem to have been linked to the radiation of land plants in quite different ways.

The almost certain origin of Amoebozoa and its major subgroups within a marine environment, leads to the question of the current lack of amoebozoan diversity in marine environment. This has been suggested to be the result of a decline associated with the Paleozoic radiation of benthic foraminiferan amoebas. These elaborately testate amoebae with fine reticulate pseudopodia probably occupy the same or similar niches as ancient marine amoebozoan amoebas [9]. This suggests that various Foraminifera outcompeted the naked amoebozoan species, perhaps by being more resistant to predation or parasitism or other rigors of the marine environment.

### 4.4: Uncertainty estimates

Although spore dating [40–42] and a number of molecular studies [43–45] place the origin of the land plant crown in the Phanerozoic (see scale bar in Figure 1), a recent molecular dating study recovered much older ages for land plants at around 568-815 Mya [54]. The latter dates are also consistent with a recent reconstruction of land colonization in arthropods from ca. 540 Mya [51]. Given our dates for an early colonization of land by amoebas, this alternative scenario would support a diversification of amoebas in synchrony with early land plant colonizers rather than before. However, this early date for land plants is still hard to reconcile with the spore fossil record.

We have relied largely on 18S rDNA for our reconstructions because these are the only molecular data available for all but a handful of taxa. However, 18S reconstructions of Amoebozoa should still be treated with caution, because weak resolution in much of the phylogeny causes uncertainty in estimating branch lengths. We have also taken a conservative approach to age reconstruction using different schemes (e.g. calibrations, models and genes). As expected these show differences, particularly when considering confidence intervals [55]. However, we feel that this conservative approach is the most comprehensive way of taking into account dating uncertainty [23], as well as discussing values in terms of temporal ranges instead of given points in time based on a single reconstruction.

A recent study finding a considerably older age for Amoebozoa of 1384-1624 Mya is due to the assignment of the oldest amoebozoan fossil to the Arcellinida [742-770 Mya; 39]. However, our reconstructions are more consistent with placing this fossil in the Tubulinea (Figure 1 [20]) or in the crown group of Lobosa [20]. Assignment of this fossil to arcellinids is also problematic because it would require assigning a marine fossil to a terrestrial clade [20,56]. A more reliable fossil that can be assigned to Arcellinida is *Centropyxis* (ca. 220 Mya, Figure 2 [57]), which again fits our temporal reconstructions better than the older estimates [58].

There are many unknowns in the study of protist diversity. Many groups are still poorly surveyed, and the predicted extent of hidden protist diversity varies widely [59]. Thus it is possible that future studies may reveal previously undetected and cryptic diversity that could modify some of the pattern of diversification we find here. However, since the signal recovered here from different dating schemes and different diversification analyses is strong, only an unprecedented and currently unpredicted diversity could radically change this pattern of co-diversification between Amoebozoa and land plants.

## Supporting Information

Figure S1 **Fossil cross-validation based on 6 proteins.** Comparison of molecularly-estimated versus known fossil ages when using molecular dates derived from single fossil calibration of trees. The use of Ascomycetes fossil on stem group is indicated as “A” and on crown group as “B” (see main text). Uniform (A and C) or Normal (B and D) distributions for node calibration were used. For each single-fossil calibration, the three resulting estimated fossil dates for the three remaining fossils nodes were compared with their known dates using the sum of the squared differences between the known and estimated dates (SSx [34]). Blue squares are SSx for each individual fossil against the three other nodes assigned to fossils. Green squares are SSx for each of the two plant fossils against the other as well as each of the two opistokont fossils against the other. Fossil ages (in million years) are indicated below their taxonomic group (fossil calibration node).
(TIF)

Table S1 **Accession numbers for the different genes used in this study.** Missing sequences were obtained from other species in the same genus, wherever possible. Sequences are taken from GenBank (www.ncbi.nih.gov), from the Joint Genome Institute (one astrerisk; www.jgi.doe.gov), and from the Genome Center at Washington University (two asterisks; genome.wustl.edu/tools/blast) and the Human Genome Sequencing Center (three asterisks; blast.hgsc.bcm.tmc.edu). New sequences are indicated in bold.
(XLSX)

Table S2 Number of species assigned to each terminal. Sources: 1) Myxogastria - http://eumycetozoa.com/data/index.php, 2) arcellinids and *Entamoeba* group - http://www.organismnames.com/, 3) dictyostelids - Romeralo et al. [16] and present study, and 4) other amoebas - http://amoeba.ifmo.ru/species.htm. Slash (/) in arcellinids, dictyostelids and Myxogastria refers to alternative species number coding (see section 2.3). One asterisk indicates (*) species number averaged to include *Hydramoeba, Parachaos, Trichamoeba, Polychaos, Deuteramoeba*; two asterisks (**) indicates inclusion of species from *Flabellula* and *Flamella*. Brackets indicate genera with species missing: one species (+1) in *Paramoeba*, one in *Pessonella*, two (+2) in *Nolandella* and *Cashia* and five (5+) in *Tecochaos, Paradermamoeba and Pseudothecamoeba* should be assigned to these taxonomic groups. *Incertaesedis* taxa are *Stygamoeba* (two species), *Stereomyxa* (two species), and *Corallomyxa* (three species).
(XLSX)

Table S3 Divergences times reconstructed for 6-proteins and 18S data set using different calibrations schemes and models. Divergence times (in million years) are for crown groups using BEAST [35], WAG model (protein) or GTR (18S), Uniform distribution and ascomycetes crown node fossil placement unless otherwise stated. AscoStem stands for ascomycetes stem node fossil placement. 95% HPD (highest posterior density) is indicated in parenthesis. NA is for Not Applicable.
(DOCX)

Nexus File S1 Nexus formatted 6-protein matrix. (NEX)

Nexus File S2 Nexus formatted 18S rDNA matrix. (NEX)
