# Supplementary figures and images for: Did Terrestrial Diversification of Amoebas (Amoebozoa) Occur in Synchrony with Land Plants?
Source: PLoS One. 2013 Sep 11;8(9):e74374. doi: 10.1371/journal.pone.0074374 (PMC3770592; doi:10.1371/journal.pone.0074374)

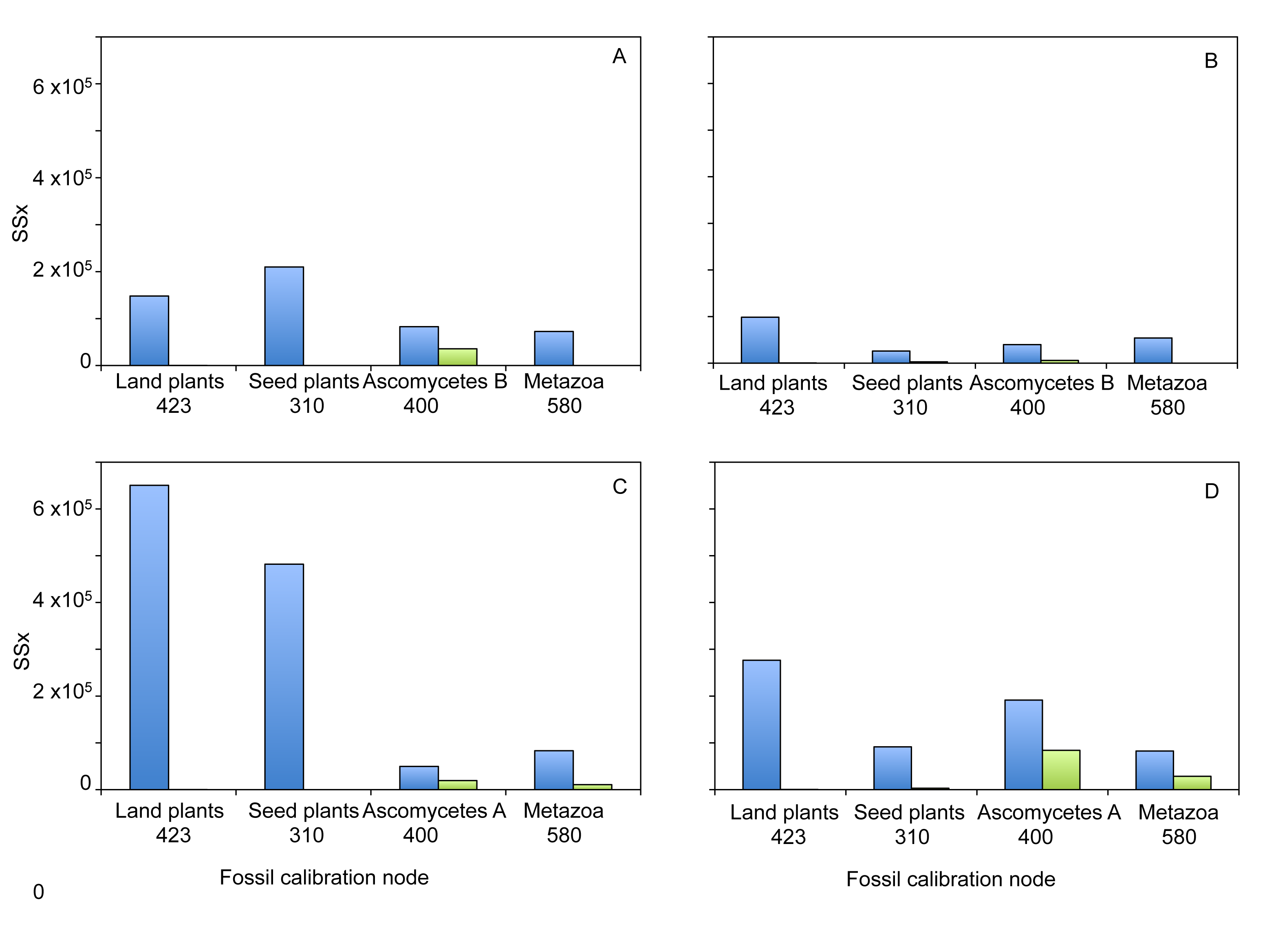

Supplement: Figure S1 — Fossil cross-validation based on 6 proteins. Comparison of molecularly-estimated versus known fossil ages when using molecular dates derived from single fossil calibration of trees. The use of Ascomycetes fossil on stem group is indicated as “A” and on crown group as “B” (see main text). Uniform (A and C) or Normal (B and D) distributions for node calibration were used. For each single-fossil calibration, the three resulting estimated fossil dates for the three remaining fossils nodes were compared with their known dates using the sum of the squared differences between the known and estimated dates (SSx [34]). Blue squares are SSx for each individual fossil against the three other nodes assigned to fossils. Green squares are SSx for each of the two plant fossils against the other as well as each of the two opistokont fossils against the other. Fossil ages (in million years) are indicated below their taxonomic group (fossil calibration node). (TIF) [file pone.0074374.s001.tif]
