# Supplementary material for: Did Terrestrial Diversification of Amoebas (Amoebozoa) Occur in Synchrony with Land Plants?
Source: PLoS One. 2013 Sep 11;8(9):e74374. doi: 10.1371/journal.pone.0074374 (PMC3770592; doi:10.1371/journal.pone.0074374)
Supplement: Table S3 — Divergence times (in million years) are for crown groups using BEAST [35], WAG model (protein) or GTR (18S), Uniform distribution and ascomycetes crown node fossil placement unless otherwise stated. AscoStem stands for ascomycetes stem node fossil placement. 95% HPD (highest posterior density) is indicated in parenthesis. NA is for Not Applicable. (DOCX) [file pone.0074374.s004.docx]

**Table S3:** Divergences times reconstructed for 6-proteins and 18S data set using different calibrations schemes and models.

| **Fossil Calibration** | **Tree Root** | **Amoebozoa** | **Mycetozoa** | **Dictyostelids** | **Arcellinids (stem)** | **Arcellinids** | **Myxogastrea** |
| --- | --- | --- | --- | --- | --- | --- | --- |
| 6-proteins 4 fossils Normal | 2039 (1129-2070) | 1437 (867-1650) | 1068 (602-1296) | 668 (331-808) | 563 (198-648) | NA | NA |
| 6-proteins 580 + 400 Normal | 1459 (899-1931) | 1125 (705-1542) | 879 (496-1220) | 500 (266-760) | 428 (161-607) | NA | NA |
| 6-proteins 580 + 400 AscoStem | 1130 (823-1474) | 949 (616-1168) | 693 (408-931) | 364 (225-590) | 220 (135-458) | NA | NA |
| 6-proteins 423 + 310 | 1748 (1202-2659) | 1208 (908-2124) | 1036 (661-1713) | 695 (367-1041) | 442 (228-817) | NA | NA |
| 6-proteins 580 + 400 + 310 Normal | 1335 (1053-1988) | 1132 (810-1583) | 962 (561-1278) | 479 (304-807) | 334 (187-619) | NA | NA |
| 6-proteins 580 + 400 + 310 Normal, AscoStem | 1110 (890-1594) | 1002 (661-1224) | 607 (455-985) | 422 (249-642) | 409 (139-498) | NA | NA |
| 18S 4 fossils | 2346 (1130-2827) | 2141 (1007-2529) | 1175 (634-1769) | 603 (240-1055) | 796 (392-1285) | 666 (249-947) | 992 (417-1293) |
| 18S 4 fossils Gamma | 2523 (1795-3000) | 2326 (1539-2747) | 1335 (906-1780) | 985 (480-1270) | 1181 (625-1608) | 888 (404-1232) | 1215 (735-1448) |
| 18S 4fossils HKY | 2597 (1730-3000) | 2454 (1556-2806) | 1626 (843-1730) | 1301 (435-1194) | 1331 (584-1691) | 979 (360-1292) | 1347 (670-1411) |
| 18S 4fossils HKY Gamma | 2401 (1822-2974) | 2246 (1641-2707) | 1192 (871-1565) | 602 (734-1464) | 1035 (651-1572) | 540 (350-1125) | 1054 (694-1321) |
| 18S 580 + 400 | 1781 (1078-2685) | 1702 (898-2408) | 1100 (669-1705) | 596 (332-1060) | 698 (435-1371) | 622 (252-1032) | 823 (471-1291) |
| 18S 580 + 400 Gamma | 1466 (804-2090) | 1435 (738-1793) | 930 (433-985) | 642 (233-746) | 853 (347-1004) | 781 (220-781) | 678 (319-746) |
| 18S 580 + 400 HKY | 1355 (1127-2376) | 1123 (1035-2108) | 857 (620-1202) | 699 (293-859) | 522 (436-1121) | 369 (286-821) | 661 (416-1090) |
| 18S 580 + 400 HKY Gamma | 1769 (964-2635) | 1753 (877-2307) | 1183 (524-1366) | 786 (334-1072) | 1039 (415-1300) | 731 (292-971) | 818 (425-1154) |

Divergence times (in million years) are for crown groups using BEAST [33], WAG model (protein) or GTR (18S), Uniform distribution and ascomycetes crown node fossil placement unless otherwise stated. AscoStem stands for ascomycetes stem node fossil placement. 95% HPD (highest posterior density) is indicated in parenthesis. NA is for Not Applicable.
